# Impact of ventriculo-cisternal irrigation on prevention of delayed cerebral infarction in aneurysmal subarachnoid hemorrhage: a single-center retrospective study and literature review

**DOI:** 10.1007/s10143-023-02241-8

**Published:** 2023-12-08

**Authors:** Motoyuki Umekawa, Gakushi Yoshikawa

**Affiliations:** 1 Department of Neurosurgery, Showa General Hospital, Tokyo, 187-8510 Japan; 2 Present Address: Department of Neurosurgery, The University of Tokyo Hospital, Tokyo, Japan

**Keywords:** Cerebral aneurysm, Delayed cerebral infarction, Intracranial pressure, Subarachnoid hemorrhage, Vasospasm, Ventriculo-cisternal irrigation

## Abstract

**Objective:**

The aim of this study was to evaluate the effectiveness of ventriculo-cisternal irrigation (VCI) in preventing vasospasms and delayed cerebral infarction (DCI) by washing out subarachnoid clots earlier after aneurysm surgery.

**Methods:**

We retrospectively identified 340 subarachnoid hemorrhage (SAH) patients with ruptured intracranial aneurysms treated with postoperative VCI at our institution between December 2010 and January 2020. As VCI therapy, a ventricular drain/cisternal drain was placed during aneurysm surgery, and lactated Ringer’s solution was used for irrigation until day 4 of SAH, followed by intracranial pressure control at 5–10 cmH_2_O until day 14.

**Results:**

The median age was 65 years (interquartile range 52–75), with 236 female patients (69%). The World Federation of Neurosurgical Societies grade distribution was as follows: grade I or II, 175 patients (51%); grade III or IV, 84 (25%); and grade V, 81 (24%). With VCI management in all patients, total vasospasm occurred in 162 patients (48%), although the DCI incidence was low (23 patients [6.8%]). Major drainage-related complications were observed in five patients (1.5%). Early surgery, performed on SAH day 0 or 1, was identified as a preventive factor against DCI occurrence (odds ratio (OR) 0.21, 95% confidence interval (CI) 0.07–0.67; *P* = 0.008), while additional surgery (4.76, 1.62–13.98; *P* = 0.005) and dyslipidemia (3.27, 1.24–8.63; *P* = 0.017) were associated with DCI occurrence.

**Conclusion:**

Managing vasospasms with VCI after SAH is considered a safe and effective method to prevent DCI. Early surgery after SAH may be associated with a decreased risk of DCI with VCI therapy.

**Supplementary Information:**

The online version contains supplementary material available at 10.1007/s10143-023-02241-8.

## Introduction

Subarachnoid hemorrhage (SAH) is one of the most severe cerebrovascular diseases, with an incidence of 1–20 patients per 100,000 [24, 38]. Approximately 85% cases are caused by intracranial aneurysm rupture [24, 38]. Its morbidity and mortality remain high, and reruptured aneurysms lead to a mortality rate of approximately 50–60% [14, 21, 37]. Although these conditions often inevitably occur at SAH onset, appropriate interventions are required to prevent rerupture.

Cerebral vasospasm followed by delayed cerebral infarction (DCI) is the most difficult condition in acute to subacute phase of SAH, rarely resulting in severe neurological impairment. Reported incidence rates are up to 70% for angiographic vasospasm and 20–40% for DCI [2, 5–7, 9, 23, 25, 30]. However, mechanism of vasospasms and standard preventative or efficacious treatments remain unclear. Historically, predominantly in Japan, SAH has been addressed through postoperative interventions involving cisternal drainage for intracranial pressure (ICP) management and ventriculo-cisternal irrigation (VCI) therapy to facilitate the expulsion of intracranial clots; these initiatives have been associated with potentially enhanced outcomes through reduced occurrences of vasospasm and mitigation of the impact of DCI [13, 15, 17, 19, 27, 34]. In line with these attempts, we have treated many patients with aneurysmal SAH (including dissecting aneurysms) at our tertiary center and adopted VCI followed by ICP control as a unified method for over 20 years. This study aimed to present the outcomes of patients treated at our institution and evaluate the efficacy of VCI and ICP control in preventing vasospasms and DCIs.

## Materials and methods

### Patient selection

Between December 2010 and January 2020, we collected data for 373 patients who developed SAH due to ruptured cerebral aneurysms (including dissecting aneurysms) and underwent open surgery from an institutional SAH database. Thirty-three patients who did not undergo VCI therapy were excluded. VCI was not performed for the following reasons: (1) Fisher group 1 SAH with no cisternal clot to wash out (*n* = 5), (2) significant angiographical vasospasm in late phase surgery after onset (*n* = 9), (3) withdrawal from treatment immediately after surgery per the request of the patient’s family (*n* =18), and (4) inability to insert a ventricular drain (VD) in an appropriate position (*n* =1). Consequently, 340 patients who underwent surgery for SAH following VCI therapy and continuous ICP control were selected. Data regarding patient-, aneurysm-, SAH-, and surgery-related factors as well as clinical outcomes, which were prospectively collected and recorded in the database, were retrospectively evaluated. Informed consent was obtained from all participants, and the study was approved by the Institutional Ethics Committee of Showa General Hospital (approval number REC-328).

### Postoperative SAH management

**Fig. 1:**
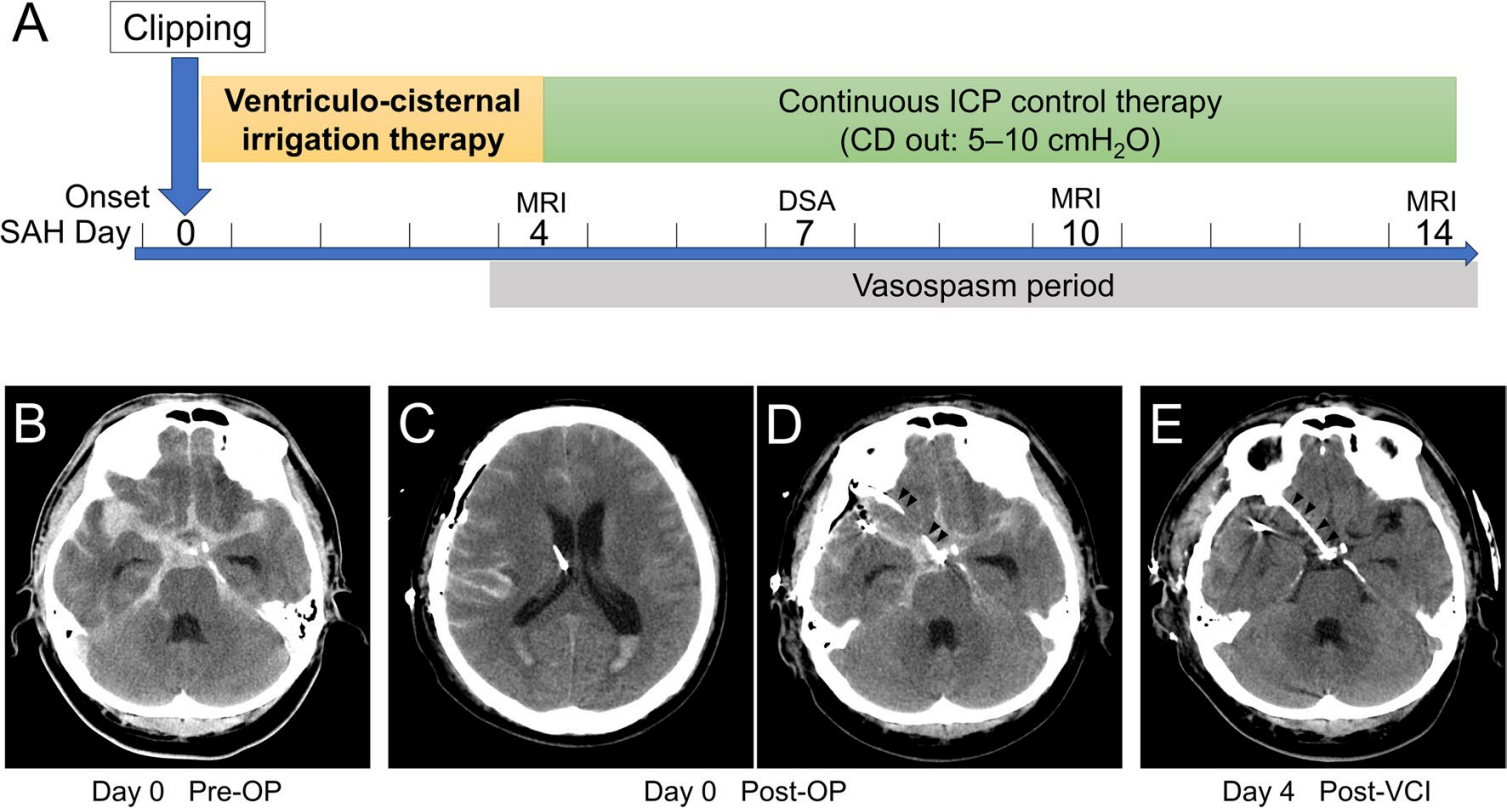
Schema showing our surgical treatment protocol and findings for a representative case. **A** Schema showing our surgical treatment protocol in the acute phase of aneurysmal subarachnoid hemorrhage (SAH), with ventriculo-cisternal irrigation (VCI) and continuous intracranial pressure (ICP) control therapy. Our approach to vasospasm prevention includes the following: (1) maximum washout of SAH by VCI until SAH day 4, (2) continuous cisternal drainage for ICP control during the vasospasm period after days 4 to 14, and (3) intensive care management and rehabilitation. **B**–**E** Representative case of SAH with a ruptured right middle cerebral artery aneurysm treated with our standardized method. **B** Preoperative computed tomography (CT) image. **C**, **D** On the day of SAH onset, aneurysm clipping is performed and ventricular (**C**) and cisternal drains (**D**; indicated by arrowheads) are placed. **E** After 4 days of VCI therapy, SAH has been completely washed out. CD cisternal drain, MRI magnetic resonance imaging, DSA digital subtraction angiography, OP operation

Figure 1A shows the treatment algorithm for aneurysmal SAH. When performing clipping or trapping surgery for SAH in Fisher group 2 or 3 with any cisternal clot, we performed VCI followed by 2 weeks of ICP control to prevent vasospasm, regardless of presence or absence of intraventricular hemorrhage. Preoperative assessment of cerebral arteries was basically performed by digital subtraction angiography (DSA); however, for patients requiring emergent surgery with increased intracranial pressure suspected, computed tomography (CT) angiography was used as an alternative. During surgery, we intraoperatively placed VD to manage cerebrospinal fluid (CSF) drainage and postoperative irrigation or drainage. VD was inserted via a frontal horn puncture in the case of surgery with supine position, or via a posterior horn puncture in the case of surgery with prone or park-bench position. Additionally, we placed a cisternal drain (CD) or lumbar drain (LD) for the CSF drainage system, which was used for postoperative management. CD was placed in the optico-carotid/precarotid cistern for the pterional approach or in the prechiasmatic cistern for the interhemispheric approach during surgery [13, 15, 17, 19, 27, 34]. In cases of surgery involving the posterior cranial fossa, LD was inserted instead of CD, postoperatively.

We administered a drop infusion system for irrigation with lactated Ringer’s solution, which was infused via VD at 20 mL/h and released from CD at the height of the forehead in the supine position. Starting on postoperative day 1, urokinase was added to the lactated Ringer’s solution at 120 U/mL to expedite clot removal through VCI. For patients with intraventricular hemorrhage, we initiated VCI inversely with infusion from CD and drainage from VD. On intraventricular hematoma resolution, we switched to regular circulation with infusion from VD and drainage from CD. After confirmation of SAH washout from CT images taken on day 4, we extracted VD and completed VCI. Thereafter, we continued CSF drainage with CD for ICP control around SAH day 14; CD was opened at the height of the forehead with the patient supine with a head-up position at 20–30°. Figure 1B–E shows representative images. During drain management, patients are generally kept at bed rest; however, for physically fit individuals, clamping drains during rehabilitation, meals, and bathroom breaks are implemented to ensure minimal disruption to activities of daily living. Cefazolin was administered intravenously during drainage as prophylaxis against meningitis.

Regarding imaging evaluation, we primarily assessed SAH distribution using CT scans until day 3. On day 4, magnetic resonance imaging (MRI) was performed to assess the early ischemic lesions and vascular conditions during the prevasospasm phase. On day 7, we performed DSA to evaluate the presence of cerebral vasospasm and aneurysms. We performed MRI to detect the presence of late-stage vasospasms and ischemic lesions on day 14. Until no evidence of clinical or angiographical vasospasms in MRI on day 14 was confirmed, close neurological examination was performed in intensive care unit. Once patients experience any neurological deficit, MRI or DSA was immediately performed to confirm status of brain and cerebral arteries.

### Evaluated outcome

A detailed investigation on VCI therapy, including the route and duration, presence of urokinase, and ICP control period during VCI therapy was conducted. The primary outcomes were the vasospasm/DCI incidence and the DCI-induced symptoms. Vasospasm was defined as any angiographical vasospasm detected by DSA or magnetic resonance angiography (MRA) with a decrease of ≥ 50% in the cerebral artery diameter relative to the diameter on preoperative DSA or CT angiography. The judgement was based on radiological findings only, regardless of any symptoms [9]. DCI was defined as symptomatic infarction identified on CT or MRI after exclusion of procedure-related infarction, with any angiographical vasospasm [39]. Procedure-related infarction was excluded by MRI on day 4 or MRI within 2 days after the initial surgery in cases where the surgery was performed after day 4. All radiological findings were independently judged by two neurosurgeons. The secondary outcome was the prospectively recorded modified Rankin scale (mRS) score at discharge. A detailed safety assessment of the complications related to drainage management was also conducted. CSF infection, defined by a prolonged period of antibiotic therapy for control of systemic inflammation accompanied by an increase in white blood cells to > 500 counts/μL in CSF, was independently assessed.

### Statistical analysis

The median and interquartile ranges (IQR) were calculated for each factor. Bivariate and multivariate logistic regression analyses were performed to determine the occurrence of vasospasms and DCI for each factor. The favorable outcome group at discharge was defined as an mRS score of 0–2, and the same analysis was conducted for this group. A *P*-value of < 0.05 was considered statistically significant. The factors used in the multivariate analysis were selected using a stepwise forward selection method with a *P*-value threshold of < 0.15. Statistical analyses were performed using JMP Pro 17 software (SAS Institute Inc., Cary, NC, USA).

### Data availability

The authors confirm that data collected for the study and analysis methods will be shared upon reasonable request from any qualified investigator.

## Results

### Characteristics of patients, SAH, and surgeries

Table 1 shows the patient demographics and SAH factors. The median age was 65 years (IQR, 52–75 years), with female patients accounting for 236 patients (69%). Hypertension was the most common comorbidity and risk factor for arterial atherosclerosis. The World Federation of Neurosurgical Societies (WFNS) grades were as follows: grade I, 72 patients (21%); II, 103 (30%); III, eight (2%); IV, 76 (22%); and V, 81 (24%). Saccular aneurysms accounted for the majority, with 319 patients (94%), while 307 patients (90%) were treated for anterior circulation aneurysms. The most common aneurysm location was the internal carotid artery-posterior communicating artery, with 88 patients (26%), followed by the anterior communicating artery, with 86 patients (25%), and the middle cerebral artery, with 74 patients (22%). Preoperative DSA was performed in 316 patients and CT angiography was performed in 24 patients for angiographical assessment.

**Table 1:** Baseline characteristics of patients with aneurysmal subarachnoid hemorrhage

	Number (%)/median [IQR]
Age, years	65 [52–75]
Female sex	236 (69%)
Comorbidities	
Hypertension	190 (56%)
Dyslipidemia	62 (18%)
Diabetes mellitus	22 (6%)
Active smoking	93 (27%)
Familial history of intracranial aneurysm	31 (9%)
Use of antithrombotic	19 (6%)
WFNS grading	
I	72 (21%)
II	103 (30%)
III	8 (2%)
IV	76 (22%)
V	81 (24%)
Aneurysm type	
Saccular	319 (94%)
Maximum dome diameter, mm	5.1 [4.0–7.6]
Dissecting	21 (6%)
Maximum length, mm	5.4 [3.0–9.3]
Location of aneurysm	
IC-Pcom	88 (26%)
Acom	86 (25%)
MCA	74 (22%)
Distal ACA	21 (6%)
IC-Ach	20 (6%)
Other anterior circulation	18 (5%)
VA	14 (4%)
BA	12 (4%)
Other posterior circulation	7 (2%)
Concomitant intracerebral hemorrhage	83 (24%)
Preoperative anisocoria/dilated pupils	45 (13%)

*ACA* anterior cerebral artery, *Acom* anterior communicating artery, *BA* basilar artery, *IC-Pcom* internal carotid artery-posterior communicating artery, *IQR* interquartile range, *MCA* middle cerebral artery, *VA* vertebral artery, *WFNS* World Federation of Neurosurgical Societies

Surgical methods included aneurysmal neck clipping in 323 patients (95%) and parental artery trapping or proximal occlusion in 17 patients (5%) (Online Resource 1). Additional techniques included bypass in 23 patients (7%) and combined decompressive craniectomy in 22 patients (6%). Ventricular drainage before aneurysm treatment was performed in 44 patients (13%). The day of surgery for aneurysm treatment was day 0 (onset of SAH) in 193 patients (57%) and day 1 in 116 patients (34%), while treatment on day 2 or later was performed in 31 patients (9%). Early surgery on day 0 or 1 was performed more frequently in the WFNS grade IV/V group (98.1% vs. 84.7% in grades I–III group; *P* < 0.001). Additional surgery, including hematoma evacuation and decompressive craniectomy, was required in 31 patients (9%).

### VCI and ICP control

VCI was performed using VD-CD irrigation in 268 patients (79%) and VD-LD irrigation in 72 patients (21%). Urokinase solution was used with VCI in 244 patients (72%). The median VCI duration was 5 days (IQR, 4–6 days), followed by a median duration of 26 days (IQR, 14–16 days) for ICP control with CD or LD (Online Resource 2).

Complications associated with drainage management were observed in 22 patients (6.5%), including five patients (1.5%) with severe complications resulting in permanent disability or requiring surgical intervention. The most common complication was bleeding around the ventricular drain in 10 patients (2.9%), two of whom (0.6%) required additional hematoma evacuation; the rest had minor bleeding without residual symptoms. Although intracranial hypotension during ICP management was observed in six patients (1.8%), only one (0.3%) required treatment, which involved hematoma evacuation for a surgical epidural hematoma. The patient fully recovered with a favorable outcome without any impairment (Online Resource 3). CSF infection was detected in 63 patients; however, no patient required prolonged hospitalization for this. Moreover, no patient developed a drainage-related abscess.

### Cerebral vasospasm and delayed cerebral ischemia: incidence and risk analysis

Of the 340 patients, 162 (47%) exhibited total vasospasms (Table 2). Of these, 139 patients (41%) had transient vasospasms, and 23 (6.8%) eventually developed into DCI. Among the 23 patients with DCI, the most common symptom was hemiparalysis in 15 (4.4%), followed by disturbed consciousness in eight (2.3%), aphasia in seven (2.1%), other cognitive function impairments in five (1.5%), and sensory disturbances in two (0.6%). The group that underwent early surgery (days 0, 1) had a DCI incidence of 5.8%, whereas the group that underwent surgery after day 2 had an incidence of 16.1% (chi-square, *P* = 0.029).

**Table 2:** Cerebral vasospasm and delayed cerebral ischemia after aneurysmal subarachnoid hemorrhage treated with surgery

	Number (%)
Total vasospasm	162 (47.6%)
Transient vasospasm	139 (40.9%)
Delayed cerebral infarction	23 (6.8%)
Motor paresis	15 (4.4%)
Consciousness disturbance	8 (2.3%)
Aphasia	7 (2.1%)
Other cognitive function disturbance	5 (1.5%)
Sensory disturbance	2 (0.6%)

Results of nominal logistic analysis of risk factors associated with total vasospasm and DCI occurrence are shown in Tables 3 and 4. In bivariate analysis, female sex, anterior circulation aneurysm, and the pterional approach were statistically significant risk factors for total vasospasm. In multivariate analysis, female sex (OR 1.64; 95% CI 1.01–2.67; *P* = 0.047) and anterior circulation aneurysm (OR 5.17; 95% CI 1.93–13.83; *P* = 0.001) remained statistically significant. Early surgery was a borderline factor for a significantly lower vasospasm risk (OR 0.47, 95% CI 0.22–1.04; *P* = 0.064). In bivariate analysis, risk factors related to DCI occurrence were dyslipidemia and postoperative additional surgery, while early surgery after SAH onset significantly reduced the risk of DCI. Multivariate analysis also identified dyslipidemia (OR 3.27; 95% CI 1.24–8.63; *P* = 0.017) and additional postoperative surgery (OR 4.76; 95% CI 1.62–13.98; *P* = 0.005) as statistically significant risk factors, with early surgery after SAH onset similarly reducing the DCI risk (OR 0.21; 95% CI 0.07–0.67; *P* = 0.008).

**Table 3:** Risk analyses related to total vasospasms after subarachnoid hemorrhage surgery under ventriculo-cisternal irrigation

	Bivariate	*P*-value	Multivariate	*P*-value
	OR [95% CI]		OR [95% CI]	
Patient factor				
Age, years (continuous)	1.00 [0.98–1.01]	0.548		
Age ≥ 65, years	0.93 [0.61–1.43]	0.749		
Female sex	1.62 [1.01–2.58]	0.045*	1.64 [1.01–2.67]	0.047*
Hypertension	1.36 [0.89–2.10]	0.158		
Dyslipidemia	1.04 [0.60–1.80]	0.897		
Diabetes mellitus	0.91 [0.38–2.17]	0.831		
Active smoking	0.69 [0.42–1.11]	0.125		
Familial history of cerebral aneurysm	1.37 [0.65–2.88]	0.402		
Use of antithrombotic	0.49 [0.18–1.32]	0.156		
SAH factor				
Dissection (vs. saccular)	0.81 [0.33–1.98]	0.651		
Anterior circulation (vs. posterior circulation)	5.86 [2.21–15.58]	< 0.001*	5.17 [1.93–13.83]	0.001*
With ICH (vs. without ICH)	1.51 [0.92–2.49]	0.104		
WFNS grade 4, 5 (vs. grades 1–3)	0.88 [0.57–1.34]	0.541		
Operative factor				
PA (vs. other approaches)	1.89 [1.15–3.15]	0.012*		
Clipping (vs. others)	1.71 [0.62–4.74]	0.301		
Bypass	1.21 [0.52–2.88]	0.653		
Decompressive craniotomy	2.01 [0.82–4.93]	0.127	2.06 [0.82–5.14]	0.122
Emergent ventricular drainage	0.90 [0.48–1.71]	0.755		
Early surgery at day 0, 1 (vs. delayed surgery after day 2)	0.47 [0.22–1.01]	0.053	0.47 [0.22–1.04]	0.064
Postoperative rerupture	1.80 [0.58–5.61]	0.313		
Postoperative additional surgery	1.84 [0.86–3.92]	0.115		

*CI* confidence interval, *ICH* intracerebral hemorrhage, *OR* odds ratio, *PA* pterional approach, *WFNS* World Federation of Neurosurgical Societies

*P values < 0.05 are considered significant

**Table 4:** Risk analyses related to delayed cerebral infarction after subarachnoid hemorrhage surgery under ventriculo-cisternal irrigation

	Bivariate	*P*-value	Multivariate	*P*-value
	OR [95% CI]		OR [95% CI]	
Patient factor				
Age, years (continuous)	1.01 [0.98–1.04]	0.560		
Age ≥ 65, years	1.31 [0.56–3.07]	0.537		
Female sex	2.19 [0.73–6.60]	0.164		
Hypertension	1.25 [0.52–2.96]	0.619		
Dyslipidemia	2.60 [1.05–6.43]	0.039*	3.27 [1.24–8.63]	0.017*
Diabetes mellitus	1.41 [0.31–6.46]	0.655		
Active smoking	0.24 [0.05–1.03]	0.055	0.24 [0.05–1.09]	0.064
Familial history of cerebral aneurysm	0.95 [0.21–4.24]	0.942		
Use of antithrombotic	2.82 [0.76–10.50]	0.122		
SAH factor				
Dissection (vs. saccular)	2.5 [0.68–9.17]	0.170		
Anterior circulation (vs. posterior circulation)	1.14 [0.25–5.09]	0.866		
With ICH (vs. without ICH)	1.39 [0.55–3.50]	0.488		
WFNS grade 4, 5 (vs. grades 1–3)	0.73 [0.31–1.75]	0.484		
Operative factor				
PA (vs. other approaches)	1.24 [0.44–3.43]	0.685		
Clipping (vs. others)	0.52 [0.11–2.43]	0.408		
Bypass	2.23 [0.61–8.13]	0.226		
Decompressive craniotomy	0.64 [0.08–4.99]	0.671		
Emergent ventricular drainage	1.46 [0.47–4.5]	0.512		
Early surgery at day 0, 1 (vs. delayed surgery after day 2)	0.32 [0.11–0.94]	0.038*	0.21 [0.07–0.67]	0.008*
Postoperative rerupture	2.65 [0.55–12.74]	0.224		
Postoperative additional surgery	4.12 [1.49–11.39]	0.006*	4.76 [1.62–13.98]	0.005*

*CI* confidence interval, *ICH* intracerebral hemorrhage, *OR* odds ratio, *PA* pterional approach, *WFNS* World Federation of Neurosurgical Societies

*P values < 0.05 are considered significant

### Overall clinical outcomes

At discharge, the performance status outcomes were as follows: favorable (mRS 0–2), 151 patients (44%); moderate (mRS 3, 4), 125 patients (37%); and severe (mRS 5, 6), 64 patients (19%) (Online Resource 4). The overall mortality rate was 5.3%. On stratification by WFNS grade, treatment for grades I and II achieved favorable outcome rates of 79% and 64% at discharge (Online Resource 5). In the grade IV and V groups, although 21% and 52%, respectively, ended up with severe outcomes (mRS 5, 6) at discharge, intermediate outcomes (mRS 3, 4) were observed for 55% and 41% patients while favorable outcomes were achieved for 24% and 7% patients, respectively (Online Resource 6). Exactly 113 patients (33%) required CSF shunt surgery because of secondary hydrocephalus, and 54 (16%) were discharged with a tracheostomy.

Bivariate and multivariate analyses of factors associated with favorable outcomes are shown in Table 5. In multivariate analysis, age ≥ 65 years (OR 0.11; 95% CI 0.06–0.22; *P* < 0.001), diabetes, anterior circulation aneurysm, concomitant ICH, WFNS grades 4 and 5, preoperative emergent VD, postoperative rerupture, and additional postoperative surgery were significantly negatively associated with a favorable outcome. Although early surgery was not associated with favorable outcomes in bivariate analysis, it was significant in multivariate analysis. Total vasospasm or DCI occurrence did not have a statistically significant negative impact on favorable outcomes at discharge.

**Table 5:** Bivariate and multivariate analyses of factors related to favorable outcomes (modified Rankin scale score 0–2)

	Bivariate	*P*-value	Multivariate	*P*-value
	OR [95% CI]		OR [95% CI]	
Patient factor				
Age, years (continuous)	0.91 [0.89–0.93]	< 0.001*		
Age ≥ 65, years	0.14 [0.09–0.23]	< 0.001*	0.11 [0.06–0.22]	< 0.001*
Female sex	0.64 [0.41–1.03]	0.065	0.54 [0.26–1.13]	0.105
Hypertension	0.60 [0.39–0.93]	0.023*		
Dyslipidemia	0.75 [0.43–1.32]	0.319		
Diabetes mellitus	0.18 [0.05–0.63]	0.007*	0.18 [0.04–0.82]	0.026*
Active smoking	1.90 [1.17–3.07]	0.009*		
Familial history of cerebral aneurysm	1.83 [0.87–3.87]	0.113		
Use of antithrombotic	0.43 [0.15–1.22]	0.111		
SAH factor				
Dissection (vs. saccular)	1.73 [0.71–4.21]	0.230		
Anterior circulation (vs. posterior circulation)	0.48 [0.23–1.01]	0.053	0.17 [0.05–0.57]	0.004*
With ICH (vs. without ICH)	0.11 [0.06–0.23]	< 0.001*	0.21 [0.08–0.54]	0.001*
WFNS grade 4, 5 (vs. grades 1–3)	0.09 [0.05–0.15]	< 0.001*	0.13 [0.06–0.26]	< 0.001*
Operative factor				
PA (vs. other approaches)	1.82 [1.09–3.03]	0.022*	2.04 [0.92–4.50]	0.079
Clipping (vs. others)	0.70 [0.26–1.85]	0.470		
Bypass	1.16 [0.50–2.71]	0.733		
Decompressive craniotomy	0.11 [0.03–0.49]	0.004*		
Emergent ventricular drainage	0.24 [0.11–0.53]	< 0.001*	0.24 [0.08–0.74]	0.012*
Early surgery at day 0, 1 (vs. delayed surgery after day 2)	0.35 [0.16–0.76]	0.008*	0.68 [0.23–2.02]	0.489
Postoperative rerupture	0.22 [0.05–1.00]	0.049*	0.12 [0.02–0.89]	0.038*
Postoperative additional surgery	0.12 [0.03–0.39]	< 0.00*	0.05 [0.01–0.22]	< 0.001*
Vasospasm factor				
Total vasospasm	0.96 [0.62–1.47]	0.836		
DCI	1.54 [0.64–3.74]	0.339		

*CI* confidence interval, *DCIs* delayed cerebral infarctions, *ICH* intracerebral hemorrhage, *mRS* modified Rankin scale, *OR* odds ratio, *PA* pterional approach, *WFNS* World Federation of Neurosurgical Societies

*P values < 0.05 are considered significant

## Discussion

This is the largest cohort study to report the management of vasospasms after aneurysmal SAH through VCI and continuous ICP control therapy. Table 6 summarizes previous reports on VCI and ICP control [13, 15, 17, 19, 27, 34, 41]. Previous studies used CD for SAH washout after surgery, and VD was subsequently introduced, leading to VCI development. These reports indicated total vasospasm rates of 15.8–57.8% and symptomatic vasospasm rates of 2.8–39.5%, which align with the present findings [13, 15, 17, 19, 27, 34, 41]. DCI occurrence is generally reported to be 20–40% [2, 5–7, 9, 23, 25, 30], and reduced occurrence (0.9–20.0%) achieved with VCI and ICP control therapy, as observed in this study, is considered favorable [13, 17, 19, 34]. Particularly noteworthy is the excellent DCI occurrence rate of 0.9% achieved by Kodama et al., who used urokinase and ascorbic acid in the irrigation fluid; this requires further verification [19]. Furthermore, systematic reviews have shown the effectiveness of intrathecal nicardipine in reducing vasospasms and DCI [4, 10] and improving the proportion of patients with favorable outcomes (mRS 0–2) [33]. In light of the above, we explored the potential effectiveness of drainage management for ICP control to administer intrathecal nicardipine on radiological vasospasm occurrence. While few previous studies have described complications related to drainage management, Kodama et al. reported drain-related bleeding and ventriculitis, with a surgical requirement rate of 1.8%, but no residual sequelae [19]. We found a low rate of severe complications associated with drainage management (1.5%). With proficiency in the management of drainage systems, VCI and continuous ICP control can be performed safely after SAH surgery.

**Table 6:** Summary of previous studies attempting to prevent cerebral vasospasm with ventriculo-cisternal irrigation and/or intracranial pressure control therapy after subarachnoid hemorrhage surgery

Author, year	Method	*n*	SAH grade	Total vasospasm	DCI	Drainage-related severe complication	Shunt surgery	Favorable outcome (mRS 0–2)	Mortality
Ito et al., 1986	ICP control	38	1–3	39.5%, symptomatic	NA	NA	NA	Good recovery in GOS, 78.9%	0
Kawakami et al., 1987	ICP control	22	2–3 (Botterell)	22.7%, symptomatic	4.5%	NA	13.6%	100%	0
Sakaki et al., 1987	ICP control	167	1–4 (H&H)	37.7%, symptomatic	16.2%	NA	NA	Return to previous activities, 58.7%	25.7%
Ogura et al., 1988	ICP control	101	1–4 (H&K)	15.8%	NA	NA	25.7%	No morbidity, 61.4%	9.9%
Inagawa et al., 1991	VCI + ICP control	140	1–4 (H&H)	57.8%	15.7%	NA	47.8%	Good recovery in GOS, 70.3%	12.6%
Kodama et al., 2000	VCI + ICP control	217	1–5 (H&K)	2.8%, symptomatic	0.9%	1.8%	38.8%	No morbidity, 80.6%	2.8%
Yamamoto et al., 2016	VCI with Mg	70	1–4 (WFNS)	62.9%	20.0%	NA	44.2%	78.5%	2.9%
Present study	VCI + ICP control	340	1–5 (WFNS)	47.6%	6.8%	1.5%	33.2%	44.4%	5.3%

*DCIs* delayed cerebral infarctions, *GOS* Glasgow Outcome Scale, *H&H* Hunt and Hess, *H&K* Hunt and Kosnik, *ICP* intracranial pressure, *mRS* modified Rankin scale, *NA* not available, *OR* odds ratio, *PA* pterional approach, *SAH* subarachnoid hemorrhage, *VCI* ventriculo-cisternal irrigation, *WFNS* World Federation of Neurosurgical Societies

Early surgery was associated with a lower DCI risk; this result aligns with the clinical significance of VCI in removing subarachnoid clots as early as possible. Previous reports have also suggested the effectiveness of early surgery in reducing vasospasms and DCI, indicating the potential benefits of early aneurysm treatment combined with VCI and ICP control in reducing DCI [12, 36]. Furthermore, early surgery reportedly improved outcomes of SAH, and its usefulness has been incorporated into the guidelines [11, 16, 24, 26, 29]. Although no relationship between early surgery and favorable outcomes was observed, our findings suggest that patients requiring early surgery could be confounded by SAH severity. Patients with radiologically visible SAH treated with surgery in the early period after onset could be good candidates for VCI.

Our cohort included 22%/24% patients with WFNS grades IV/V, respectively, indicating a population with higher severity than that in previous reports on VCI [13, 17, 19, 34], which may explain the lower proportion of favorable outcomes (44%). While vasospasm or DCI occurrence was not associated with a favorable outcome, DCI did not show a significant positive effect on outcomes because of the low DCI incidence due to early surgery and management with VCI. We evaluated physical status at discharge using an assessment, potentially increasing favorable outcomes. Considering the above, compared with previously reported rates, favorable or intermediate outcomes, interpreted as outcomes where a bedridden status is prevented, were achieved in 48% patients, even in the WFNS grade V group [1, 18, 20, 31, 35, 40]. This finding could potentially imply the effectiveness of VCI therapy. Although less invasive treatment with stereotactic cisternal drainage has resulted in DCI prevention and favorable outcomes [32], in recent years, especially after clazosentan trials, discussions regarding vasospasm have progressed to revolve around a multifactorial etiology, including early brain injury, for worse outcomes [3, 8, 22]. Therefore, continuous ICP control through CD can result in improvements in prognosis. Further studies are needed to determine whether this management approach can improve the long-term performance status of patients. Against this background, a previous study showed that spreading depolarization, considered a cause of early brain injury and worse outcomes, can be triggered by spike-like increases in ICP [28]. Therefore, continuous ICP control through CD can result in improvements in prognosis. Further studies are needed to determine whether this management approach can improve the long-term performance status of patients.

## Limitations

This study had several limitations. First, this was a single-center, single-arm, retrospective study without a comparative control group. Therefore, these results could not be single out VCI as an influencing factor for reduction of DCIs. In addition, this study is limited in its generalizability as it exclusively includes patients who underwent surgery for aneurysmal SAH, excluding those who underwent endovascular treatments. Second, only patients who received VCI treatment in this study were classified as Fisher grade 2 or 3, indicating a selection bias. Thus, the analysis may have been performed in a population more prone to vasospasms. Third, the clinical treatment outcome was assessed at discharge, and not all follow-up data after discharge were available. A long-term prospective comparable study is required to evaluate the effectiveness of this treatment.

## Conclusion

VCI and continuous ICP control after surgery for SAH due to a ruptured aneurysm are considered effective and safe, reducing the DCI rate to 6.8%. Early surgery following SAH onset was particularly effective in preventing DCI.

## Supplementary information

ESM 1 (DOCX 22 kb)

ESM 2 (DOCX 16 kb)

ESM 3 (DOCX 16 kb)

ESM 4 (DOCX 16 kb)

ESM 5 (PNG 532 kb)

High Resolution (TIF 1288 kb)

ESM 6 (DOCX 17 kb)

## Data Availability

Available upon reasonable request.
