# Supplementary material for: Impact of ventriculo-cisternal irrigation on prevention of delayed cerebral infarction in aneurysmal subarachnoid hemorrhage: a single-center retrospective study and literature review
Source: Neurosurg Rev. 2023 Dec 8;47(1):6. doi: 10.1007/s10143-023-02241-8 (PMC10703947; doi:10.1007/s10143-023-02241-8)
Supplement: Supplementary file 1 — (DOCX 22 kb) [file 10143_2023_2241_MOESM1_ESM.docx]

***Neurosurgical Review***

Impact of ventriculo-cisternal irrigation on prevention of delayed cerebral infarction in aneurysmal subarachnoid hemorrhage: a single-center retrospective study and literature review

Motoyuki Umekawa, Gakushi Yoshikawa

Correspondence:

Motoyuki Umekawa

Department of Neurosurgery,

Showa General Hospital, Tokyo 187-8510, Japan.

Email: [moto.umekawa@gmail.com](mailto:moto.umekawa@gmail.com)

ORCID: 0000-0002-7722-9861

**Online Resource 1.** Details of the surgical procedure for ruptured aneurysms

|  | Number (%) |
| --- | --- |
| Clipping | 323 (95%) |
| Trapping/proximal ligation | 17 (5%) |
| Surgical approaches |  |
| Pterional | 254 (75%) |
| Interhemispheric | 64 (19%) |
| Suboccipital | 20 (6%) |
| Subtemporal | 2 (1%) |
| Adjunctive surgical procedure |  |
| Bypass | 23 (7%) |
| Decompressive craniotomy | 22 (6%) |
| Emergent ventricular drainage | 44 (13%) |
| Day of surgery |  |
| Day 0 | 193 (57%) |
| Day 1 | 116 (34%) |
| Day 2–4 | 23 (7%) |
| Day 5 or later | 8 (2%) |
| Postoperative rupture | 13 (4%) |
| Postoperative additional surgery | 31 (9%) |
