# Supplementary material for: Impact of ventriculo-cisternal irrigation on prevention of delayed cerebral infarction in aneurysmal subarachnoid hemorrhage: a single-center retrospective study and literature review
Source: Neurosurg Rev. 2023 Dec 8;47(1):6. doi: 10.1007/s10143-023-02241-8 (PMC10703947; doi:10.1007/s10143-023-02241-8)
Supplement: Supplementary file 2 — (DOCX 16 kb) [file 10143_2023_2241_MOESM2_ESM.docx]

***Neurosurgical Review***

Impact of ventriculo-cisternal irrigation on prevention of delayed cerebral infarction in aneurysmal subarachnoid hemorrhage: a single-center retrospective study and literature review

Motoyuki Umekawa, Gakushi Yoshikawa

Correspondence:

Motoyuki Umekawa

Department of Neurosurgery,

Showa General Hospital, Tokyo 187-8510, Japan.

Email: [moto.umekawa@gmail.com](mailto:moto.umekawa@gmail.com)

ORCID: 0000-0002-7722-9861

**Online Resource 2.** Details of ventriculo-cisternal irrigation and intracranial pressure control therapy for aneurysmal subarachnoid hemorrhage treated with surgery

|  | Number (%)/Median [IQR] |
| --- | --- |
| Ventriculo-cisternal irrigation |  |
| VD-CD | 268 (79%) |
| VD-LD | 72 (21%) |
| Use of urokinase irrigation | 244 (72%) |
| Duration of VCI, days | 5 [4–6] |
| Duration of ICP control by CSF drainage, days | 15 [14–16] |

CD, cisternal drain; CSF, cerebrospinal fluid; IA, intra-arterial injection; ICP, intracranial pressure; IQR, interquartile range; IV, intra-venous injection; LD, lumbar drainage; PTA, percutaneous transluminal angioplasty; VCI, ventriculo-cisternal irrigation; VD, ventricular drain
