# Supplementary material for: Impact of ventriculo-cisternal irrigation on prevention of delayed cerebral infarction in aneurysmal subarachnoid hemorrhage: a single-center retrospective study and literature review
Source: Neurosurg Rev. 2023 Dec 8;47(1):6. doi: 10.1007/s10143-023-02241-8 (PMC10703947; doi:10.1007/s10143-023-02241-8)
Supplement: Supplementary file 3 — (DOCX 16 kb) [file 10143_2023_2241_MOESM3_ESM.docx]

***Neurosurgical Review***

Impact of ventriculo-cisternal irrigation on prevention of delayed cerebral infarction in aneurysmal subarachnoid hemorrhage: a single-center retrospective study and literature review

Motoyuki Umekawa, Gakushi Yoshikawa

Correspondence:

Motoyuki Umekawa

Department of Neurosurgery,

Showa General Hospital, Tokyo 187-8510, Japan.

Email: [moto.umekawa@gmail.com](mailto:moto.umekawa@gmail.com)

ORCID: 0000-0002-7722-9861

**Online Resource 3.** Drainage-related complications

|  | All complications | Asymptomatic complications | Severe complications |
| --- | --- | --- | --- |
| Total | 22 (6.5%) | 17 (5.0%) | 5 (1.5%) |
| CD-related hemorrhage | 2 (0.6%) | 1 (0.3%) | 1 (0.3%) |
| VD-related hemorrhage | 10 (2.9%) | 8 (2.4%) | 2 (0.6%) |
| Compression due to inappropriate CD location | 3 (0.9%) | 3 (0.9%) | 0 (0%) |
| Intracranial hypotension due to overdrainage | 6 (1.8%) | 5 (1.5%) | 1 (0.3%) |
| Drainage-related abscess | 1 (0.3%) | 0 (0%) | 1 (0.3%) |

CD, cisternal drain; VD, ventricular drain
