# Supplementary material for: Impact of ventriculo-cisternal irrigation on prevention of delayed cerebral infarction in aneurysmal subarachnoid hemorrhage: a single-center retrospective study and literature review
Source: Neurosurg Rev. 2023 Dec 8;47(1):6. doi: 10.1007/s10143-023-02241-8 (PMC10703947; doi:10.1007/s10143-023-02241-8)
Supplement: Supplementary file 4 — (DOCX 16 kb) [file 10143_2023_2241_MOESM4_ESM.docx]

***Neurosurgical Review***

Impact of ventriculo-cisternal irrigation on prevention of delayed cerebral infarction in aneurysmal subarachnoid hemorrhage: a single-center retrospective study and literature review

Motoyuki Umekawa, Gakushi Yoshikawa

Correspondence:

Motoyuki Umekawa

Department of Neurosurgery,

Showa General Hospital, Tokyo 187-8510, Japan.

Email: [moto.umekawa@gmail.com](mailto:moto.umekawa@gmail.com)

ORCID: 0000-0002-7722-9861

**Online Resource 4.** Performance status at discharge after aneurysmal subarachnoid hemorrhage treated with surgery

| Modified Rankin Scale score at discharge | Number (%) |
| --- | --- |
| 0 | 63 (19%) |
| 1 | 35 (10%) |
| 2 | 53 (16%) |
| 3 | 48 (14%) |
| 4 | 77 (23%) |
| 5 | 46 (14%) |
| 6 | 18 (5%) |
| Discharge destination | Number (%) |
| Home | 92 (27%) |
| Rehabilitation hospital | 192 (56%) |
| Nursing care facilities | 34 (10%) |
| Others | 4 (1%) |
