# Supplementary figures and images for: Impact of ventriculo-cisternal irrigation on prevention of delayed cerebral infarction in aneurysmal subarachnoid hemorrhage: a single-center retrospective study and literature review
Source: Neurosurg Rev. 2023 Dec 8;47(1):6. doi: 10.1007/s10143-023-02241-8 (PMC10703947; doi:10.1007/s10143-023-02241-8)

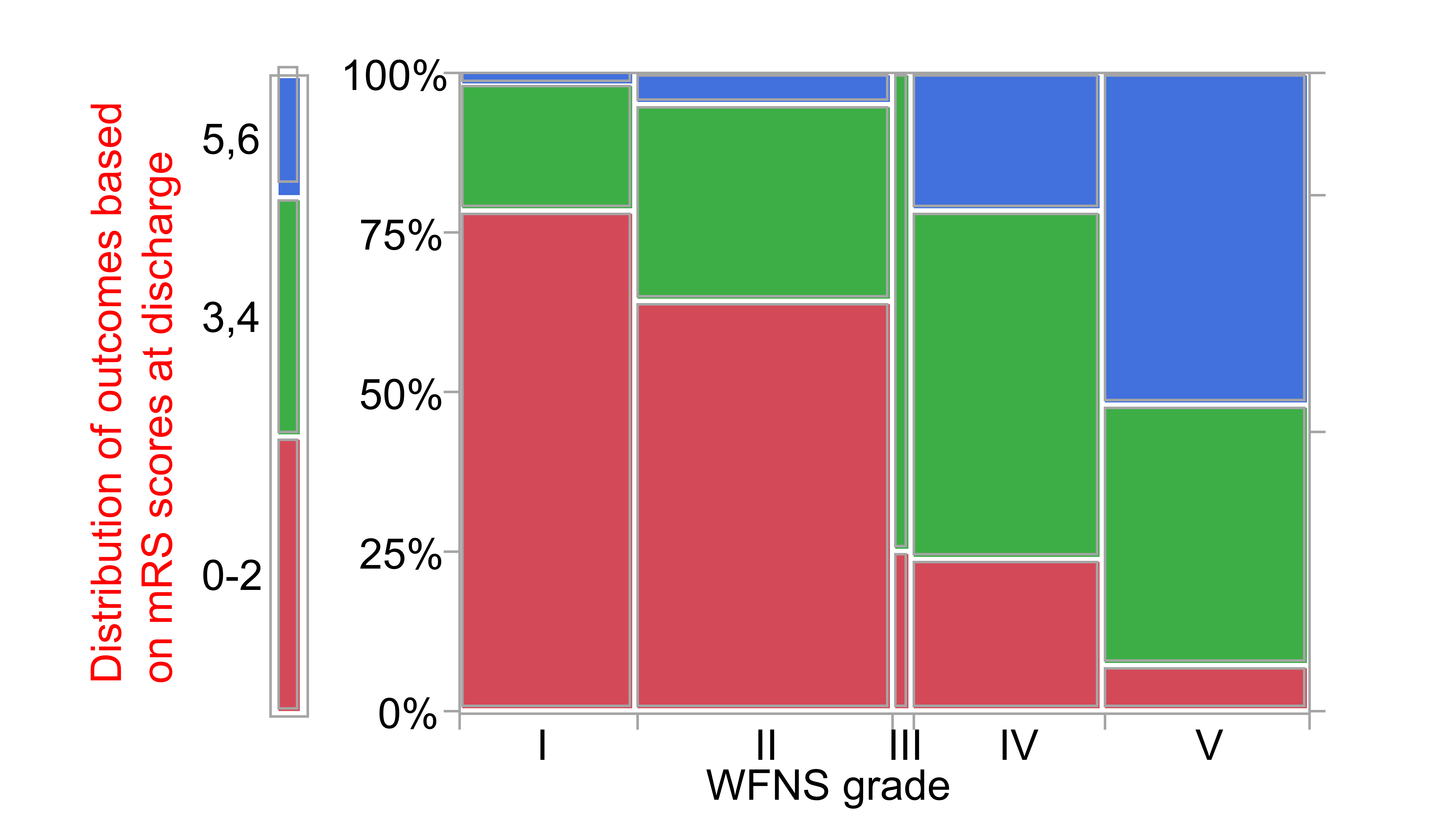

Supplement: Supplementary file 5 — (PNG 532 kb) [file 10143_2023_2241_Fig2_ESM.png]

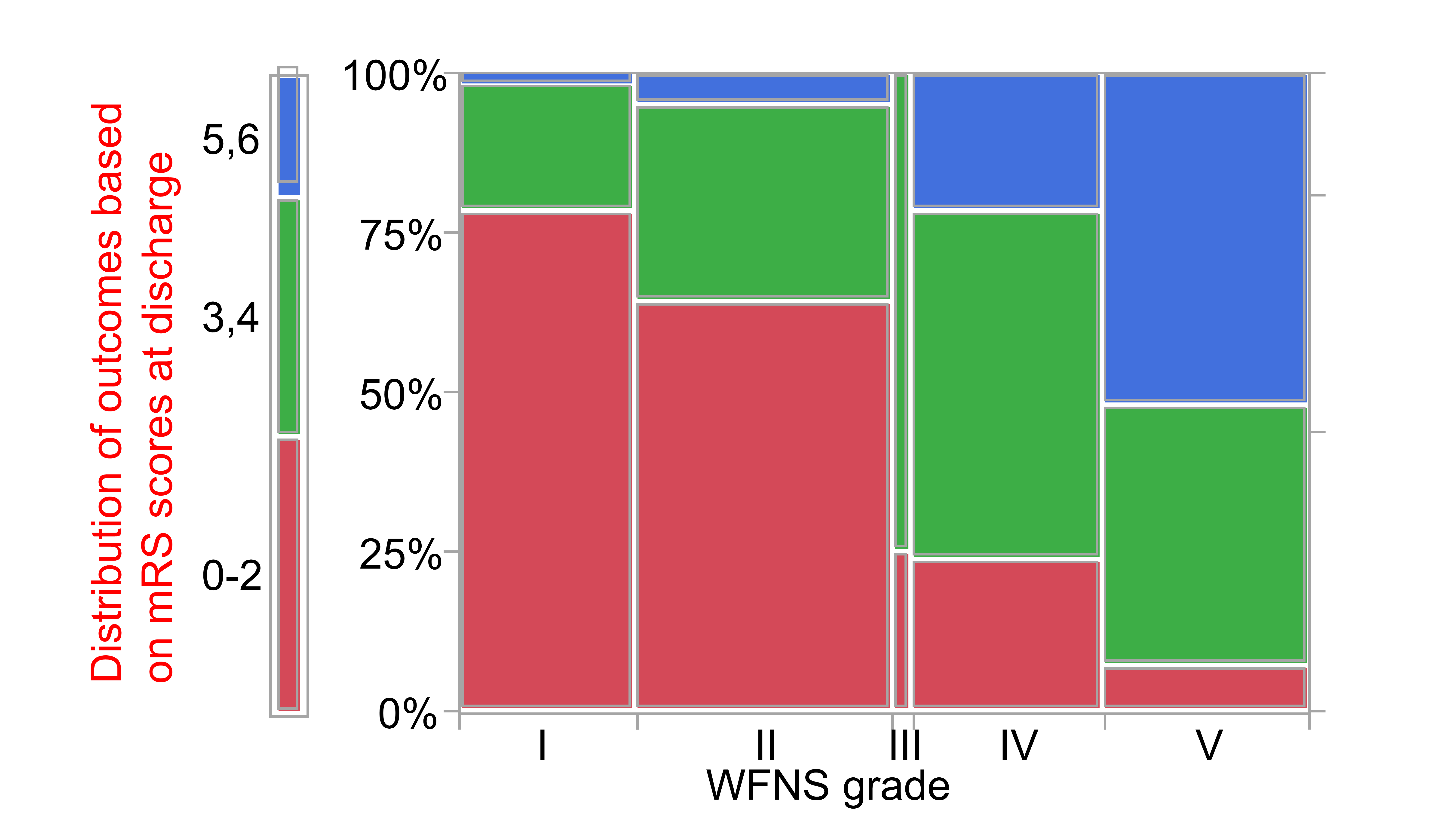

Supplement: Supplementary file 6 — High Resolution (TIF 1288 kb) [file 10143_2023_2241_MOESM5_ESM.tif]
