# Supplementary material for: Impact of ventriculo-cisternal irrigation on prevention of delayed cerebral infarction in aneurysmal subarachnoid hemorrhage: a single-center retrospective study and literature review
Source: Neurosurg Rev. 2023 Dec 8;47(1):6. doi: 10.1007/s10143-023-02241-8 (PMC10703947; doi:10.1007/s10143-023-02241-8)
Supplement: Supplementary file 7 — (DOCX 17 kb) [file 10143_2023_2241_MOESM6_ESM.docx]

***Neurosurgical Review***

Impact of ventriculo-cisternal irrigation on prevention of delayed cerebral infarction in aneurysmal subarachnoid hemorrhage: a single-center retrospective study and literature review

Motoyuki Umekawa, Gakushi Yoshikawa

Correspondence:

Motoyuki Umekawa

Department of Neurosurgery,

Showa General Hospital, Tokyo 187-8510, Japan.

Email: [moto.umekawa@gmail.com](mailto:moto.umekawa@gmail.com)

ORCID: 0000-0002-7722-9861

**Online Resource 6.** Performance status at discharge stratified by the World Federation of Neurosurgical Societies grade after aneurysmal subarachnoid hemorrhage treated with surgery

|  | Modified Rankin Scale score at discharge | | | | | | |
| --- | --- | --- | --- | --- | --- | --- | --- |
| WFNS grade | 0 | 1 | 2 | 3 | 4 | 5 | 6 |
| 1 | 30 (41.7%) | 13 (18.1%) | 14 (19.4%) | 7 (9.7%) | 7 (9.7%) | 0 | 1 (1.4%) |
| 2 | 13 (18.1%) | 17 (16.5%) | 21 (20.4%) | 16 (15.5%) | 15 (14.6%) | 3 (2.9%) | 2 (1.9%) |
| 3 | 0 | 0 | 2 (25.0%) | 1 (12.5%) | 5 (62.5%) | 0 | 0 |
| 4 | 4 (5.3%) | 4 (5.3%) | 11 (14.5%) | 12 (15.8%) | 29 (38.2%) | 12 (15.8%) | 4 (5.3%) |
| 5 | 0 | 1 (1.2%) | 5 (6.2%) | 12 (14.8%) | 21 (25.9%) | 31 (38.3%) | 11 (13.6%) |

WFNS, World Federation of Neurosurgical Societies
